# The value of lncRNA *FENDRR* and *FOXF1* as a prognostic factor for survival of lung adenocarcinoma

**Published:** 2017-10-27

**Authors:** Antonio Herrera-Merchan, Marta Cuadros, Maria Isabel Rodriguez, Sandra Rodriguez, Raul Torres, Marcos Estecio, Isabel F. Coira, Claudia Loidi, Monica Saiz, Pedro Carmona-Saez, Pedro P. Medina

**Affiliations:** ^1^ Centre for Genomics and Oncological Research, PTS Granada, Centro Pfizer - Universidad de Granada - Junta de Andalucía de Genómica e Investigación Oncológica (GENYO), Granada, Spain; ^2^ Department of Biochemistry and Molecular Biology I, University of Granada, Granada, Spain; ^3^ Department of Biochemistry and Molecular Biology III and Immunology, University of Granada, Granada, Spain; ^4^ Molecular Cytogenetics Group, Human Cancer Genetics Program, Spanish National Cancer Research Centre-CNIO, Madrid, Spain; ^5^ Department of Epigenetics and Molecular Carcinogenesis, UT MD Anderson Cancer Center, Houston, TX, USA; ^6^ Pathological Anatomy, University Hospital Cruces, University of Pais Vasco, Spain

**Keywords:** lncRNA, FENDRR, FOXF1, lung cancer, methylation

## Abstract

It is increasingly evident that non-coding RNAs play a significant role in tumour development. However, we still have a limited knowledge of the clinical significance of long non-coding RNAs (lncRNAs) in lung cancer. The *FENDRR* is a long coding RNA (also named *FOXF1-AS1*) located in the vicinity of the protein-coding gene *FOXF1* at 16q24.1 chromosomal region. The present study aimed to define the clinic pathological significance of the long-non-coding RNA *FENDRR* in lung adenocarcinomas. *FENDRR* expression measured by quantitative PCR was found significantly downregulated (p<0.001) in lung adenocarcinoma samples in comparison with their normal adjacent tissues (n=70). RNA *in situ* hybridization (RNA-FISH) corroborated independently the down-regulation of *FENDRR*. Interestingly, the expression of *FENDRR* correlated positively (p<0.001) with the expression of its protein-coding neighbor gene *FOXF1*. Additionally, *FOXF1* expression was also found downregulated in adenocarcinomas compared to normal samples (p<0.001) and its expression was significantly correlated with overall survival alone (p=0.003) or in combination with *FENDRR* expression (p=0.01). In conclusion, our data support that *FENDRR* and *FOXF1* expression is decreased in lung adenocarcinoma and should be considered as new potential diagnostic/prognosis biomarkers.

## INTRODUCTION

Lung cancer is the most lethal cancer in most developed countries. Around half of the patients diagnosed with lung cancer die within one year of diagnosis and the 5-year survival rates are less than 18% [1]. One of the main barriers to further progress in lung cancer therapy is the lack of effective biomarkers. However, research efforts are currently being diverted toward identifying new cancer biomarkers that would improve outcome in these patients. Growing research evidence have determined an unexpected relevant role of long non coding RNAs (lncRNAs) in tumor development [2, 3] that have opened new opportunities for the discovery of effective tumor biomarkers that can improve patient diagnosis and prognosis.

LncRNAs are transcripts with more than 200 nucleotides of length that have a limited or no protein-coding capacity [4-6]. Previous studies have demonstrated that lncRNAs have multifunctional roles in different cellular processes and in tumorigenesis by participating in both oncogenic and tumour suppressing pathways [7, 8]. Although the specific function of most lncRNAs is still in investigation, they can regulate a large fraction of targets working either in trans or in cis [9].

The *FENDRR* lncRNA (also named *FOXF1-As1*) and its neighbor protein-coding gene *FOXF1* are located in the vicinity, with less than 2 kb of difference, at the long arm of chromosome 16, although they are transcribed from opposite strand [10]. *FENDRR* and *FOXF1* are expressed in specific adult tissues as bladder, colon, esophagus, lung, prostate, stomach and small intestine [11]. Pioneer studies have assessed the impact of *FENDRR* in cancer. Initially, *FENDRR* was firstly reported to be downregulated in gastric cancer compared with normal gastric cells and the low *FENDRR* expression was related to poor prognosis [12]. Recently, in lung cancer, a lncRNA expression profiling identified to *FENDRR* as one of the lncRNAs were differentially expressed between cancer and the adjacent normal tissues [13]. Additionally, also in lung cancer, *FENDRR* expression was associated with tumour migration and metastasis [14]. However, the overall contributions of *FENDRR* to lung cancer remain to be investigated.

In this study, we examined the expression levels of *FENDRR* and *FOXF1* in lung cancer patients through qRT-PCR. We found *FENDRR* and *FOXF1* expression was downregulated in lung adenocarcinomas compared to adjacent normal lung tissues and that *FENDRR* mRNA expression levels correlated positively with *FOXF1* expression. Interestingly, we found that *FENDRR* and *FOXF1* had also a prognosis value since their high expression values were associated with a better clinical outcome in lung adenocarcinomas.

## RESULTS

### *FENDRR* and *FOXF1* expression is lost in lung adenocarcinomas

**Figure 1:**
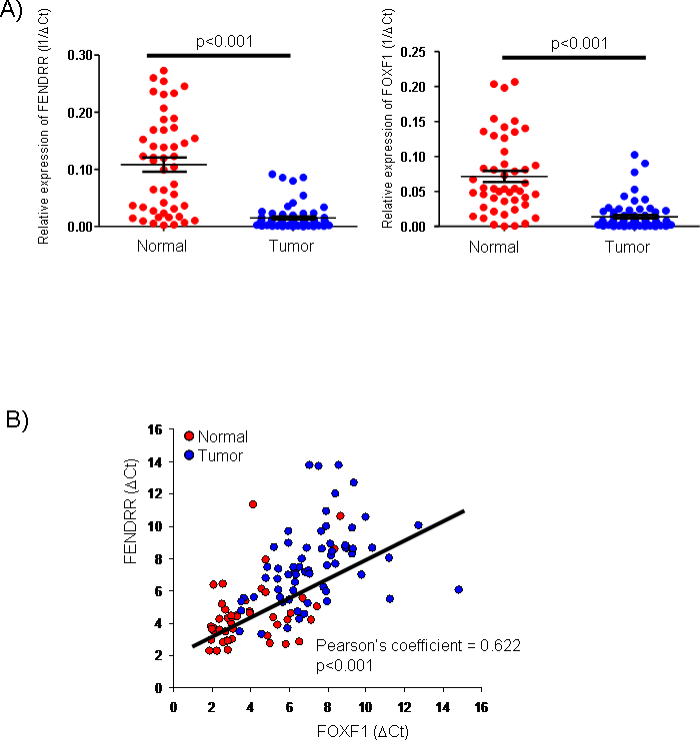
*FENDRR* and *FOXF1* mRNAs are significant biomarkers of lung adenocarcinoma (LUAD). **(A)** *FENDRR* and *FOXF1* expression was analyzed by qRT-PCR in 70 LUAD and 70 normal lung tissues. *FENDRR* and *FOXF1* were significantly downregulated in lung adenocarcinomas versus their adjacent normal lung tissues. **(B)** The expression levels of *FENDRR* and *FOXF1* was positive correlated in each other in lung tumor and adjacent normal lung tissues (Pearson's coefficient = 0.622, p<0.001).

*FENDRR* and *FOXF1* expression was analyzed by qRT-PCR in 70 lung adenocacinomas (LUAD) and their adjacent normal lung tissues. As shown in Figure 1A, the expression of *FENDRR* and *FOXF1* was significantly lower in the tumors than in adjacent matched normal tissue samples (p <0.001 in both cases). In almost all cases, *FENDRR* and *FOXF1* expression levels in tumors were lower than those observed in normal samples, with a median in *FENDRR* expression levels of 0.008 and with a range value from 0,000034 to 0.273. While *FOXF1* expression in tumor samples ranged from 0,00007 to 0.207, with a median of 0.0059. Interestingly, *FENDRR* expression levels showed a significant positive correlation with *FOXF1* (Figure 1B).

To corroborate these results, we used external datasets from GEO (Gene Expression Omnibus) and TCGA (http://methhc.mbc.nctu.edu.tw). Importantly, several and independent cancer datasets showed a significant positive correlation between *FENDRR* expression and *FOXF1* RNA expression (Table 1).

**Table 1:** Data from different expression profiles of cancer available in GEO and TCGA database

Study	Authors	Tissue	Type Cancer	Nº Samples	FENDRR expression	p-value	FOXF1 expression	p-value
GSE18842	Sanchez-Palencia, 2011	Lung	LUSC	45	-1.14	<0.001	-3.26	0.002
			LUAD	45			-2.52	0.015
TCGA-LUAD	doi:10.7908/C18G8K47	Lung	LUAD	546	-2.31	<0.001	-2.02	<0.001
TCGA-LUSC	doi:10.7908/C1XW4J7P	Lung	LUSC	548	-2.26	<0.001	-2.13	<0.001
GSE19804	Lu TP, 2010	Lung	NSCLC	120	-1.16	<0.001	-2.01	<0.001
GSE31210	Okayama H, 2012	Lung	LUAD	246	-2.16	<0.001	-2.45	<0.001
GSE3268	Wachi S, 2005	Lung	LUSC	10	ND	ND	-1.85	<0.001
GSE55945	Arredouani MS, 2009	Prostate	PCA	21	-0.83	0.032	-1.08	0.021
GSE24514	Alhopuro P, 2010	Colon	CRC	49	ND	ND	-1.52	<0.001

The *FENDRR* and *FOXF1* expression value of the tumors was normalized by the non-tumoral samples. CRC: Colorectal cancer; LUAD: Lung Adenocarcinoma; LUSC: Lung Squamous Cancer; NSCLC: Non Small Cell Lung Cancer; PCA: Prostate Cancer.

### *FENDRR* and *FOXF1* expression: lung cancer cell lines

*FENDRR* and *FOXF1* expression levels were quantified by qRT-PCR in 5 human lung cancer cell lines (A549, H441, H661, H838 and H460), displaying similar trend than in primary tumors. Actually, four of them (A549, H441, H661 and H838) have lost most of the *FENDRR* and *FOXF1* expression. Just H460, expressed *FENDRR* and *FOXF1* at measurable levels by qPCR (Supplementary Figure 1). According to our results, the *in silico* analysis of *FENDRR* and *FOXF1* expression from Cancer Cell Line Encyclopedia (CCLE) database [15] showed a significant positive correlation between *FENDRR* and *FOXF1* in 1036 cancer cell lines (p<0.001) (Supplementary Figure 2A), and in particular 116 NSCLC cancer cell lines (p<0.00001). Interestingly, a human cell line derived from normal embryonic lung tissue (HLFA) displayed the highest levels *FOXF1* and *FENDRR* (Supplementary Figure 2B).

### *FENDRR* expression by RNA-FISH

**Figure 2:**
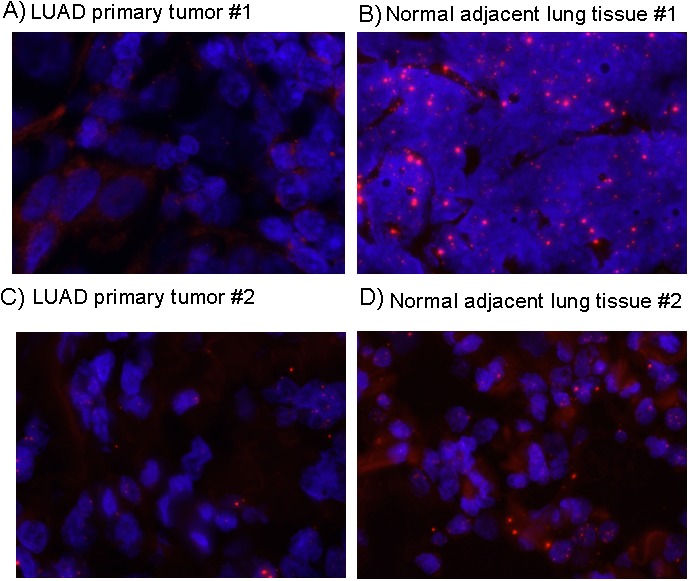
*FENDRR* expression detection by RNA-FISH in lung cancer and normal lung. RNA *FENDRR* fluorescence in situ hybridization (RNA-FISH) in two representative pairs of primary lung adenocarcinoma **(A, C)** and adjacent normal tissue **(B, D)**. Red: *FENDRR* probe, bluet: DAPI.

To confirm its association with lung adenocarcinoma, using an independent method to qPCR, we examined *FENDRR* expression by RNA-FISH on 6 paraffin embedded LUAD and matched healthy tissues. When present, *FENDRR* staining was nuclear and cytoplasmic. RNA-FISH analysis showed that *FENDRR* was differently expressed in tumour and normal cells (Figure 2), and the qPCR analysis displayed their expression is lost in tumors.

Together, these data supported the conclusion that *FENDRR* could be a new biomarker for lung cancer.

### High *FENDRR* and *FOXF1* expression is associated with better clinical outcome in lung cancer

**Figure 3:**
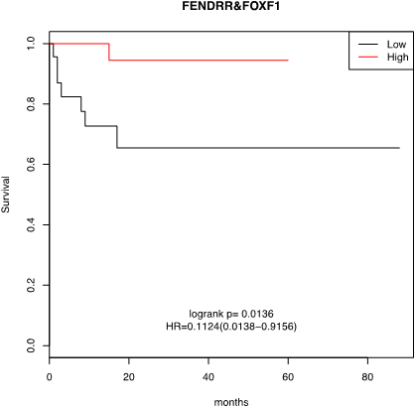
Kaplan-Meier survival analysis for the relationship between survival time and *FENDRR* and *FOXF1* signature in lung adenocarcinomas. Higher *FENDRR* and *FOXF1* expression was associated with a better overall survival for patients with lung cancer. The survival function is defined as the probability of surviving at least to the time determined in x-axis (in months).

Because *FENDRR* and *FOXF1* could influence outcome, we studied whether the expression levels of *FENDRR* and FOXF1 genes could predict the treatment response and/or survival of LUAD patients. No significant differences were found in the distribution of common clinical variables such as age, gender, pathological stage, lymph node status, type of treatment, relapse, and smoking in the groups of LUAD cases showing low or high expression of *FENDRR* or *FOXF1* (Table 2). As show in Figure 3, Kaplan-Meier curves of cases in the combined high-*FENDRR/FOXF1* expression group (n=64) showed a significant better survival times than patients with tumors with lower expression (p=0.01). These results are corroborated using larger dataset of lung cancer patients was in silico analyzed by the Kaplan–Meier software (KM plotter.com) [16]. Three microarray data sets were available from GEO under accession number GSE31210, GSE30219 and GSE3141, which contained 226, 142 and 76 samples of lung cancer patients respectively. Tumors were separated into two groups based on *FENDRR* expression. Low expression of *FENDRR* was associated with shorter survival of patients in these three datasets (hazard ratio [HR], 0.08-0.067; 95% CI, 0.01 to 0.96; p<0.05). The results were summarized in Supplementary Figure 5. We confirmed the association with overall survival for *FENDRR* and also found an association with survival probability.

**Table 2:** Association between *FENDRR* and *FOXF1* expression and clinicopathological features (only available for 64 patients)

Clinicopathologic characteristics	FENDRR p-value	FOXF1 p-value
**Gender**	0.61	0.67
Male (45)		
Female (19)	
Age (y)	0.42	0.38
<66 (36)		
>66 (28)		
Pathological stage (T)	0.61	0.11
I (12)		
II (37)		
III (8)		
IV (1)		
Unknow (6)		
Regional lymph nodes (N)	0.56	0.74
0 (29)		
1 (10)		
2 (7)		
Unknow (18)		
Adjuvant treatment	0.65	0.12
None (29)		
Chemotherapy (18)		
Chemotherapy & Radiotherapy (11)		
Radiotherapy (3)		
Unknow (3)		
Relapse	0.95	0.79
Yes (24)		
No (38)		
Unknow (2)		
Smoking at the time of the diagnosis	0.50	0.09
Yes (56)		
No (5)		
Unknow (2)		

*FENDRR* and *FOXF1* are considered as continues variable.

### Mutational analysis of *FENDRR* and *FOXF1* in lung cancer

**Figure 4:**
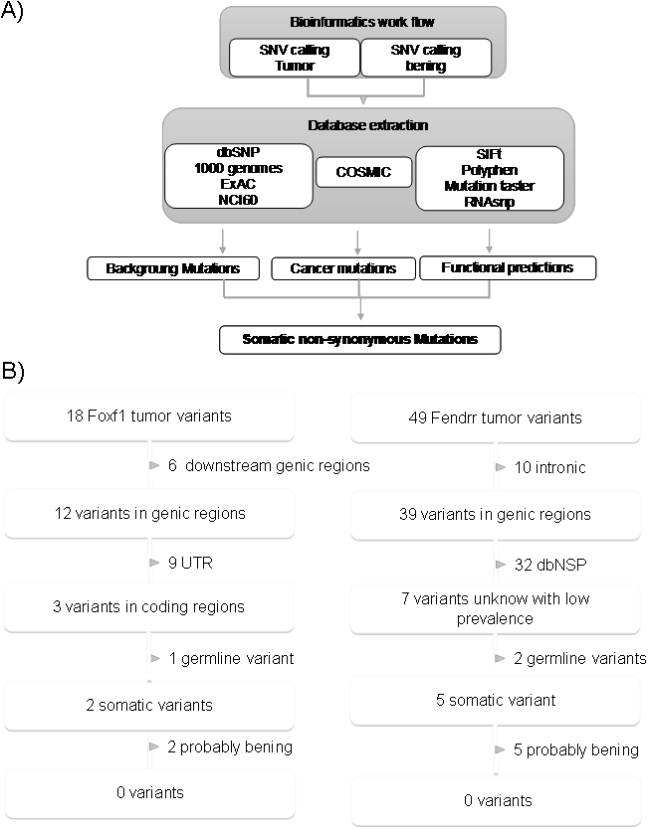
Identification process of somatic variants in *FENDRR* and *FOXF1*. **(A)** Schematic of bioinformatics SNV detection workflow. **(B)** Extraction of functionally relevant somatic mutations for *FOXF1* and *FENDRR* in lung cancer. Variants were filtered for annotation in dbSNP, 1000genomes, ExAC, NCI60, somatic and functionally impairment. From dbSNP or the 1000 genomes variants with frequencies above 1% were excluded.

To gain additional insights into the inactivation mechanism of *FENDRR* and *FOXF1*, we decided to study the mutation status of the genes. For this purposed we sequenced these two genes in 27 pairs of matched tumor and normal of lung tissues. We found 49 and 18 single nucleotide variations in *FENDRR* and *FOXF1* genes respectively. Since it is difficult to determine the functional repercussion of the mutations, especially in long non-coding genes, we filtered the data obtained following the analysis workflow described in the Figure 4A. None of these alterations were considered with as probable cause of the loss of expression of *FENDR* and *FOXF1* we observed in LUAD because they were located in the introns, or in untranslated regions (in case of FOXF1), were reported as dbSNP, somatic or predicted as being (Figure 4B) described in detail.

### Methylation status of *FENDRR* and *FOXF1*

**Figure 5:**
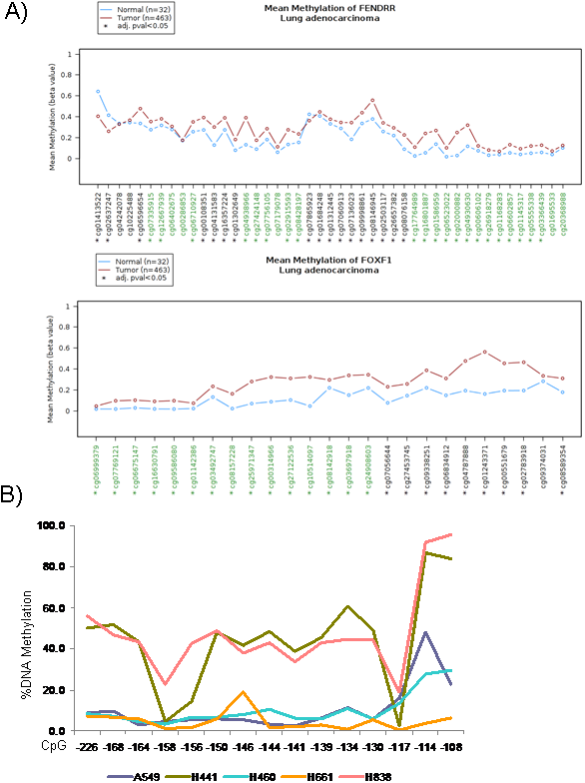
Epigenetic inactivation of *FENDRR* and *FOXF1* in lung adenocarcinoma. **(A)** Graphic obtained from Wanderer data base (http://maplab.imppc.org/wanderer/) of all CpGs island of *FENDRR* and *FOXF1* and their percentage of DNA methylation in lung cancer (medium of 463 samples, brown) and normal lung (medium of 32 samples, blue). In x-axis the cg# indicates the position of the CpG island. ^*^: indicates differences statistically significant between normal and tumor samples. **(B)** Percentage of DNA Methylation in the *FOXF1* promoter in 5 lung cancer cells lines by bisulphite sequencing. Hypermethylation of *FOXF1* promoter was detected in 2 of 5 cell lines (H441 and H838).

Another well-known mechanism of gene silencing is promoter hypermethylation. To determine the involvement this epigenetic mechanism of silencing for *FENDRR* and *FOXF1*, firstly we analyzed the *in silico* results from The Cancer Genome Atlas database [17] using the Wanderer web tool [18]. The methylation status of the promoter CpG islands of *FENDRR* and *FOXF1* was significantly different in tumor versus normal tissue. Approximately 84% and 96% of CpG islands were differentially methylated for *FENDRR* and *FOXF1* respectively although mean methylation level difference between tumor and normal was even sensibly higher in the *FOXF1* case (Figure 5A). For this reason, we decided to analyze the methylation status of the *FOXF1* promoter experimentally by pyrosequencing of bisulfite-modified DNA and MSP analysis in A549, H441, H661, H838 and H460 human lung cancer cell lines. Sequence analysis detected hypermethylation of *FOXF1* promoter in 2 of 5 cell lines (H441 and H838) (Figure 5B), indicating that hypermethilation of *FOXF1* promoter could mediate the downregulation of *FOXF1* in lung cancer. Interestingly, treatment with demethylating agent 5-aza-deoxycytidine (5-Aza) of lung cancer cell lines didn't produce any change in methylation of *FOXF1* promoter (Supplementary Figure 3).

Next, we searched for the presence of promoter hypermethylation by MSP analysis at *FOXF1* in 10 lung adenocarcinoma samples and their adjacent normal tissues (Supplementary Figure 4), aberrant promoter hypermethylation was detected in 2 of 10 lung adenocarcinoma patients (2/10, 20%).

### *FENDRR* and *FOXF1* functional relationship

**Figure 6:**
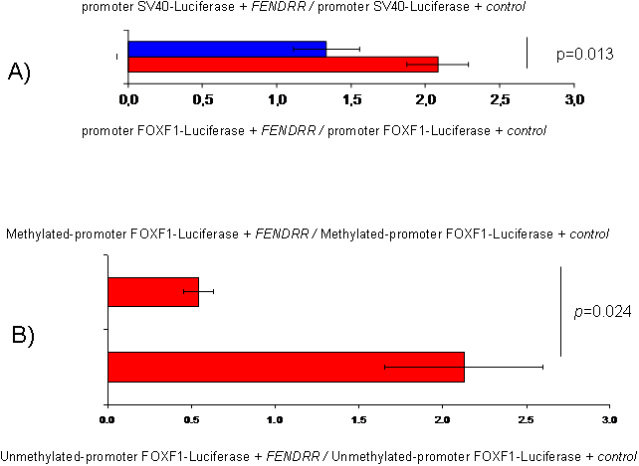
*FENDRR* and *FOXF1* relationship determined by luciferase assays in the A549 cell line. **(A)** Normalized luciferase expression comparing the luciferase reporter under *FOXF1* promoter with and without methylation after adding *FENDRR*. Error bars represent standard deviation. **(B)** Control: Normalized luciferase expression comparing the reporters under the *FOXF1* promoters with and without methylation after *FENDRR* transfection. *FENDRR*: expression plasmid of *FENDRR*. Control: expression plasmid where *FENDRR* has been substituted by GFP.

Recent studies have showed that many lncRNAs can alter gene expression via dsDNA:RNA triplex formation and recruit protein complexes [19-21]. To explore this possibility and analyze *FENDRR* and *FOXF1* functional relationship we developed luciferase assays. Interestingly, after transfecting a plasmid expressing *FENDRR* in A549 cells we observed an 56% increment in the normalized luciferase expression when compared the reporter under *FOXF1* promoter with the reporter under SV40 promoter that was used as reference (p=0.013) (Figure 6A). However, these differences where not appreciated when the FOXF1 promoter is methylated (Figure 6B, p=0.024) indicating that the binding of *FENDRR* to *FOXF1* promoter could be sensitive to the methylation status of the DNA.

## DISCUSSION

Multiple lines of evidence increasingly link mutations and dysregulations of lncRNAs to diverse human diseases including cancer [22]. Recent progress suggests that the involvement of lncRNAs in human diseases could be far more prevalent than previously appreciated [23]. Therefore, the identification and investigation of cancer associated lncRNAs is critical for understanding the roles of lncRNAs in the carcinogenesis and may have clear clinical importance in lung cancer [23].

*FENDRR* and *FOXF1* expression levels are relatively high in healthy lung tissue, where, with a median of 14 and 31 RPKM (Reads per kilo base per million of mapped reads) (n=320) respectively ranks between the top-5 tissues with more expression (Supplementary Figure 6) [11]. In the present study, we have investigated *FENDRR* and *FOXF1* expression by qRT-PCR and RNA-FISH assays in lung adenocarcinoma patients. The results indicated that expression of *FENDRR* and *FOXF1* in lung cancer tissues was significantly lower than those observed in adjacent normal tissues, suggesting the potential of using *FENDRR* and *FOXF1* to successfully distinguish cancerous from normal tissues. Furthermore, we found *FENDRR* and *FOXF1* significantly associated to survival. And patients with low levels *FENDRR* and *FOXF1* expression had a significantly poorer overall survival compared with patients expressing high levels of these genes.

Based on these results, *FENDRR* and *FOXF1* could be postulated to be as potential tumor suppressor genes. The mechanisms of inactivation of these genes in different tumor types have not been yet established. Alterations in the coding sequence have rarely been found in lung cancer [24, 25], indicating that mechanism of *FENDRR* and *FOXF1* inactivation could be, among others, loss of heterozygosity (LOH) and promoter hypermethylation. In our dataset, the mutation is not frequently found in *FENDRR* or *FOXF1* mutation. However, the methylation analysis showed a relatively high incidence of *FOXF1* methylation among lung cancer patients and lung tumoral cell lines. Mitchell SM et al. [26] and PK Lo et al. [27] also found an important percentage of *FOXF1* methylation in colorectal cancer and breast cancer. Our results, however, showed that *FOXF1* expression could by silenced by promoter methylation also in lung cancer.

LncRNAs have been reported to affect the expression of neighboring genes positively or negatively. Based on mRNA expression analysis that revealed a positive correlation between *FENDRR* and *FOXF1* for lung cancer, we explored the possible functional relationship between *FENDRR* and *FOXF1* using luciferase assays. Our results indicated that *FENDRR* expression enhanced the activity of *FOXF1* promoter significantly. This could be possible if *FENDRR* is able to bind to promoter region of *FOXF1* directly and enhance *FOXF1* transcription. Actually, using *in silico* bioinformatics analysis we suggested previously that *FENDRR* could regulate expression of *FOXF1* forming a triplex-helix DNA:RNA structure [28]. Interestingly, we observed that DNA methylation abrogated the changes mediated by *FENDRR* suggesting that the binding of *FENDRR* to *FOXF1* promoter could be sensitive to the methylation status of the promoter (Figure 6B).

We are still far to understand the roles of *FOXF1* and *FENDRR* in the cancer development but there are suggestive functional data that can explain the tumor-suppressor features we have observed. *FOXF1*, a member of the forkhead box family of transcription factors that has been previously shown to be critical for lung development, homeostasis, and injury responses. It was observed that FOXF1 is induced upon DNA damage in a p53-dependent manner whereas the inactivation of *FOXF1* stimulated cell invasion and migration [29]. More unknown is the role of *FENDRR* in tumor development. A recent report showed that mouse embryos lacking *Fendrr* displayed upregulation of several transcription factors controlling heart development [30]. The authors of this report showed that *Fendrr* binds to cancer related complexes like both PRC2 and TrxG/MLL, suggesting that it acts as modulator of chromatin signatures that define gene activity and to contribute to cell identity and differentiation, a feature lost during the tumor development.

In summary, this study identifies *FENDRR* and *FOXF1* as novel potential tumour suppressor genes in lung cancer and that *FENDRR* acts by binding to unmethylated *FOXF1* promoter. *FENDRR* and *FOXF1* gene expression were predicted for overall survival, where patients with higher levels of *FENDRR* and *FOXF1* expression had better prognosis. Thus, much more work is still required to determine the detailed mechanisms it functions in lung cancer and the potentiality of *FENDRR* and *FOXF1* as therapeutic targets for lung cancer.

## MATERIALS AND METHODS

### Tissue collection

The study was approved by the Ethics Committee (CEI Granada), Department of Health, Government of Andalucía and from Basque Foundation for Health Innovation and Research, Spain. Participants provided written consent in accordance with institutional and national guidelines; consent procedure was also approved by the Ethics Committee. Seventy tumours samples from lung adenocarcinoma (LUAD) were taken from primary malignant lung tumours, as well as their adjacent non-tumoural tissue, based on macroscopic examination, by a trained pathologist.

### Reverse transcription

RNA obtained was reverse transcribed in the presence of 5 mM MgCl_2_, 1X PCR Buffer II, 1 mM dNTPs, 25 ul MuLV Reverse Transcriptase, 1 ul RNA Ribonuclease inhibitor, 2.5 μM Random hexamers in a final reaction volume of 20 μl. DNA-ase was using during the protocol to avoid gDNA amplificacion. Reactions were carried out at 42°C for 30 minutes in a Gene Amp PCR system 9600 (PE Applied Biosystems), followed by a 10-minute step at 99°C to denature the enzyme, and then by cooling to 4°C.

### Quantitative RT-PCR analysis

A SYBR Green quantitative real-time PCR (qRT-PCR) was carried out to quantify the expression levels of *FENDRR* lncRNA and *FOXF1* protein-coding RNA. All PCRs were performed using the ABI prism 7900 system (Applied Biosystems) under the conditions recommended by the manufacturers. All experiments were performed in triplicate and the mean of triplicates was used. The gene expression was determined using comparative threshold cycle (Ct). Afterward, the mean threshold cycle value of GAPDH as a reference gene was subtracted from the mean threshold cycle value of the target genes (*FENDRR* and *FOXF1*) to obtain ΔCt, and ΔΔCt values of each sample were calculated from the corresponding Ct values. qPCR primers are indicated in the Supplementary Table 1. Finally, target/internal control gene was calculated using the formula expression ratio R = 2^-DCt. Differences in gene expression groups were estimated using Student's t-tests.

### RNA fluorescence in situ hybridization (RNA-FISH)

*FENDRR* FISH was performed on thin (approximately 4 μm thick) tissue sections mounted on positively charged slides (SuperFrost, Thermo Scientific). The QuantiGene ViewRNA Assays (Affimetrix) was used following the manufacturer's instructions. Briefly, the method consists of sample permeabilization in protease to allow target accessibility, followed by target hybridization with a specific *FENDRR* probe. After the hybridization, the RNA signals are amplified via a series of sequential hybridization steps. Finally, the slides are washed in cold 2xSSC, 1xSSC and 0.5xSSC and mounted in Vectashield with 1 ug/ml DAPI (Vector) prior to microscopy analyses. *FENDRR* was visualized using a standard fluorescence microscope and images were captured using a CCD camera (Photometrics SenSys camera) connected to a PC running the Zytovision image analysis system (Applied Imaging Ltd., UK) with focus motor and Z stack software. All slides were examined for *FENDRR* ISH signals in morphologically intact cells and scored manually by S.R.

### Mutational status of *FENDRR* and *FOXF1*

A custom capture gene panel with *FENDRR* and *FOXF1* coding sequences and 150 bp of upstream and downstream flanking sequences was designed using NimbleGen's SeqCap EZ Choice Library capture system (Roche NimbleGen, Inc, Madison, WI). Libraries were prepared using 300 ng of genomic DNA. After appropriate quality controls, libraries were pooled and captured according to NimbleGen's SeqCap EZ Choice Library capture protocols. Captured libraries were multiplexed twenty per cartridge and sequenced to generate 2x150 bp paired-end reads using NextSeq 500/550 Mid Output v2 kit (300 cycles) on a NextSeq 500 sequencer (Illumina, San Diego, CA).

### Cell lines and culture conditions

Cell lines A549, H441, H661, H838 and H460 were purchased from American Type Culture Collection (ATCC) and were cultured based on the conditions suggested by ATCC. They were cultivated at 37ºC and a 5% CO_2_ atmosphere in RPMI medium supplemented with 10% fetal bovine serum, penicillin, streptomycin and amphotericin B. Cell line cultures were tested regularly for mycoplasma infection using Venor® GeM-qEP qPCR kit. Cells were used to low passes after defrosting.

### Methylation analysis

100 ng to 500 ng of genomic DNA was treated with sodium bisulfite using the EZ DNA Methylation-Gold Kit (Zymo Research, Irvine, CA) according to the manufacturer's protocol. The samples were eluted in 20 ul of M-Elution Buffer, and 1 ul to 3 ul were used for each PCR reaction. Both bisulfite conversion and subsequent pyrosequencing analysis were done at the DNA Methylation Analysis Core, The University of Texas M.D. Anderson Cancer Center.

PCR primers for pyrosequencing methylation analysis of the genomic area proximal to the transcription start site of *FOXF1* were designed using the Pyromark Assay Design SW 1.0 software (Qiagen, Hilden, Germany). Pyrosequencing and MSP primers are indicated in the Supplementary Table 1. In brief, a sequencing primer was identified within 1 to 5 base pairs near the CpG sites of interest, with an annealing temperature of 40±5 ºC. After that, forward and reverse primers are identified uspstream and downstream to the sequencing primer. Optimal annealing temperatures for each of these primers were tested using gradient PCR. Controls for high methylation (SssI-treated DNA), low methylation (WGA-amplified DNA) and no-DNA template were included in each reaction. PCR reactions were performed in a total volume of 15 μl, and the entire volume was used for each pyrosequencing reaction as previously described [31]. Briefly, PCR product purification was done with streptavidin-sepharose high-performance beads (GE Healthcare Life Sciences, Piscataway, NJ), and co-denaturation of the biotinylated PCR products and sequencing primer (3.6 pmol/reaction) was conducted following the PSQ96 sample preparation guide. Sequencing was performed on a PSQ HS 96 system (Biotage AB, Uppsala, Sweden) with the PyroMark Gold Q96 CDT Reagents (Qiagen, Hilden, Germany) according to the manufacturer's instructions. The degree of methylation was calculated using the Pyro-Q CpG 1.0.9v software (Biotage AB, Uppsala, Sweden). The DNA obtained from FFPE tissues was investigated using methylation-specific PCR according to standard protocols, with minor modifications [32].

### Treatment with 5-aza-deoxycytidine

5-aza-CdR was dissolved in water to a final concentration of 10 mg/ml and stored in aliquots at −80°C. The 5-aza-decoxycytidine treatment was optimized to establish a working concentration, using a range from 1 to 50 μM. The cells were exposed to 5-aza-CdR for 2 days to allow the drug to be incorporated into DNA at 10 μM concentration. Tissue culture medium was changed every day for both control and treated cells, to maintain the drug stability during treatment.

### Luciferase reporter assay

The dual-luciferase Reporter Assay System (Promega) was used to monitor the interaction of *FENDRR* on *FOXF1* promoter. A cloned segment of 1.7 kb DNA from the region between *FENDRR* and *FOXF1* was introduced in vector pGL3-control vector. All SV40 coding sequences are removed. Transfections on A549 lung cancer cell line were performed using TransIT-X2® Dynamic Delivery System (Mirus Bio LLC) per triplicate.

### Generation of lncRNA *FENDRR* plasmid

We purchased the clone HLUNG2006614 (pME18SFL3 vector) from Biological Resource Center (NBRC), National Institute of Technology and Evaluation (NITE). Next, it was subcloned into the viral vector pLVC-AcGFP-N1-PGK-PURO removing AcGFP region.

### CpG methylation of the plasmid vector

pGL3-promoter vector was incubated 8 hours at 37 °C with and without methyl donor S-adenosylmethionine 10 uM (NEB) and M.SssI (30 unit). DNA was purified by using a gel extraction kit and eluted in 30 μl of H2O.

### Statistical analysis

Student t-test was used to compare means of continuous variables, and Chi-square or 2-sided Fisher exact test were chosen for categorical variables. Correlations of gene expression levels with factors were performed using two-sample Welch or Wilcoxon rank sum tests. Data analyses were carried out with the SPSS statistical software, version 15.0 (SPSS Inc., Chicago, IL) and R. Using Anderson-Darling normality test implemented in the “nortest” R package we determined that FENDERR and FOXF1 datasets, are normaly distributed.

For survival analysis, high or low expression of FENDRR and FOXF1 was established for each patient if their expression levels were higher or lower than the median, respectively. Overall survival of patients with high and low expression was then compared using Kaplan-Meier and log-rank test.

Relative risks and their 95% CI were estimated by Cox proportional hazards regression.

## SUPPLEMENTARY MATERIALS FIGURE



## Abbreviations

lncRNA: Long non-coding RNA
qRT-PCR: Quantitative real-time polymerase chain reaction
RNA-FISH: RNA in situ hybridization
LUAD: Lung adenocarcinoma
Ct: Threshold cycle
SSC: Saline-sodium citrate buffer
UTR: Untranslated region
ATCC: American Type Culture Collection
RPMI: Roswell Park Memorial Institute medium
5-Aza: 5-aza-deoxycytidine
NBRC: Biological Resource Center
NITE: National Institute of Technology and Evaluation
FFPE: Formalin-fixed paraffin-embedded
LUSC: lung cell squamous cancer
CCLE: Cancer Cell Line Encyclopedia
TCGA: The Cancer Genome Atlas
MSP: Methylation-Specific PCR
GFP: Green fluorescent protein
OS: Overall survival
HR: Hazard ratio.
