# Supplementary material for: The value of lncRNA FENDRR and FOXF1 as a prognostic factor for survival of lung adenocarcinoma
Source: Oncotarget. 2017 Oct 27;11(13):1172–85. (PMC7138163)
Supplement: Supplementary file 1 [file oncotarget-11-1172-s001.pdf]

The value of lncRNA *FENDRR* and *FOXF1* as a prognostic factor for survival of lung adenocarcinoma

SUPPLEMENTARY MATERIALS

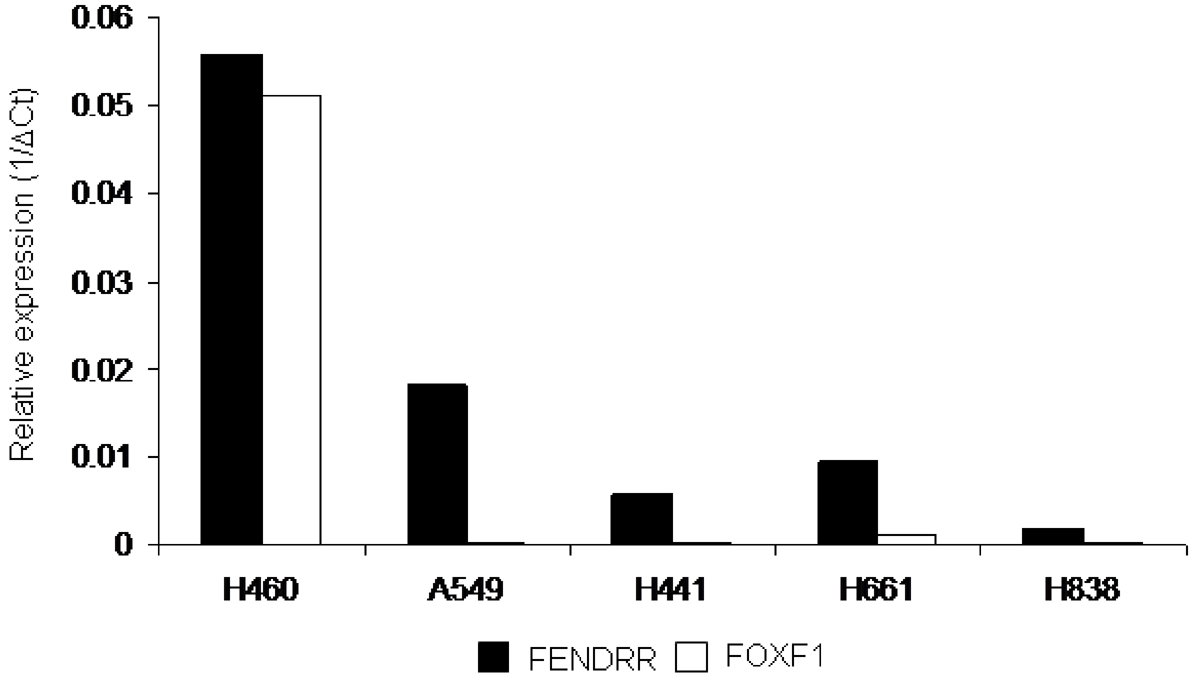

Supplementary Figure 1: *FENDRR* and *FOXF1* mRNA expression on lung cancer cell lines. The mRNA expression levels of Fendrr and Foxf1 was determined by qRT-PCR on 5 lung cancer cell lines (A549, H441, H661, H838 and H460) available in ours lab. The H460 cell line expressed *FENDRR* and *FOXF1*.

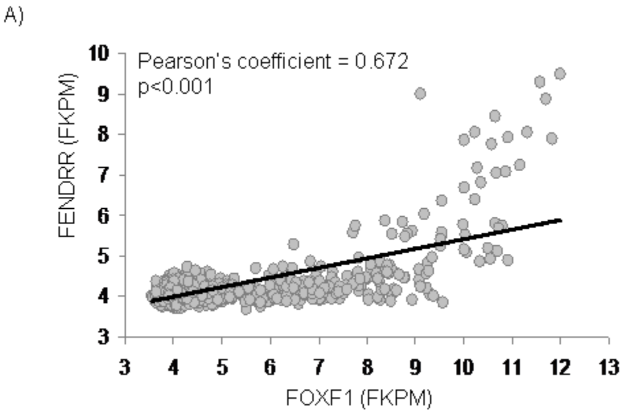

Supplementary Figure 2: In silico analysis of *FENDRR* and *FOXF1* expression from Cancer Cell Line Encyclopedia (CCLE) database. **(A)** Correlation of *FENDRR* and *FOXF1* expression levels in 1036 cancer cells lines. **(B)** Correlation of *FENDRR* and *FOXF1* expression levels in Non Small Lung Cancer cell lines (n=116). HLFA, a human cell line derived from normal embryonic lung tissue, showed the highest expression levels of *FENDRR* and *FOXF1*. *FPKM* (fragments per kilobase of transcript per million mapped reads).

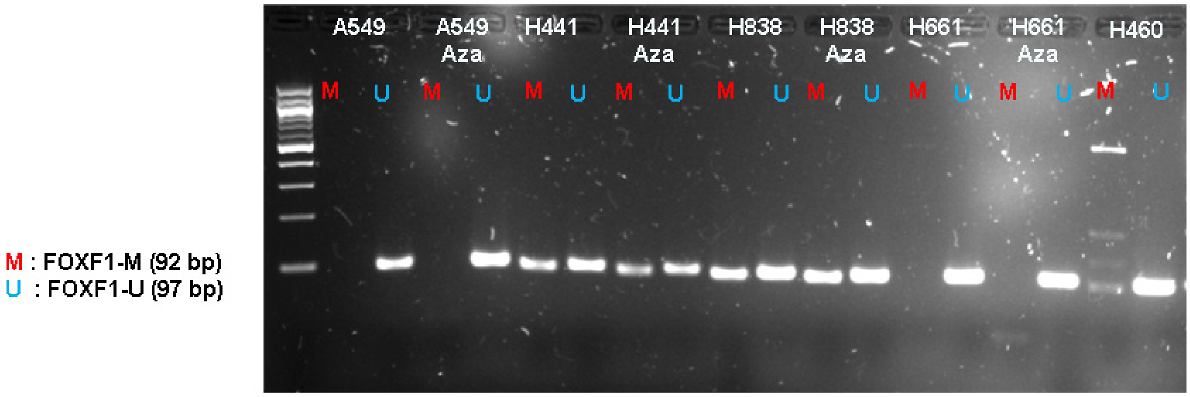

Supplementary Figure 3: Methylation-specific PCR (MSP) assay for the detection of *FOXF1* promoter hypermethylation in 4 lung cancer cells lines treated with or without 5-aza-deoxycytidine (5-Aza). H460 cell line was used as control of *FENDRR* and *FOXF1* expression.

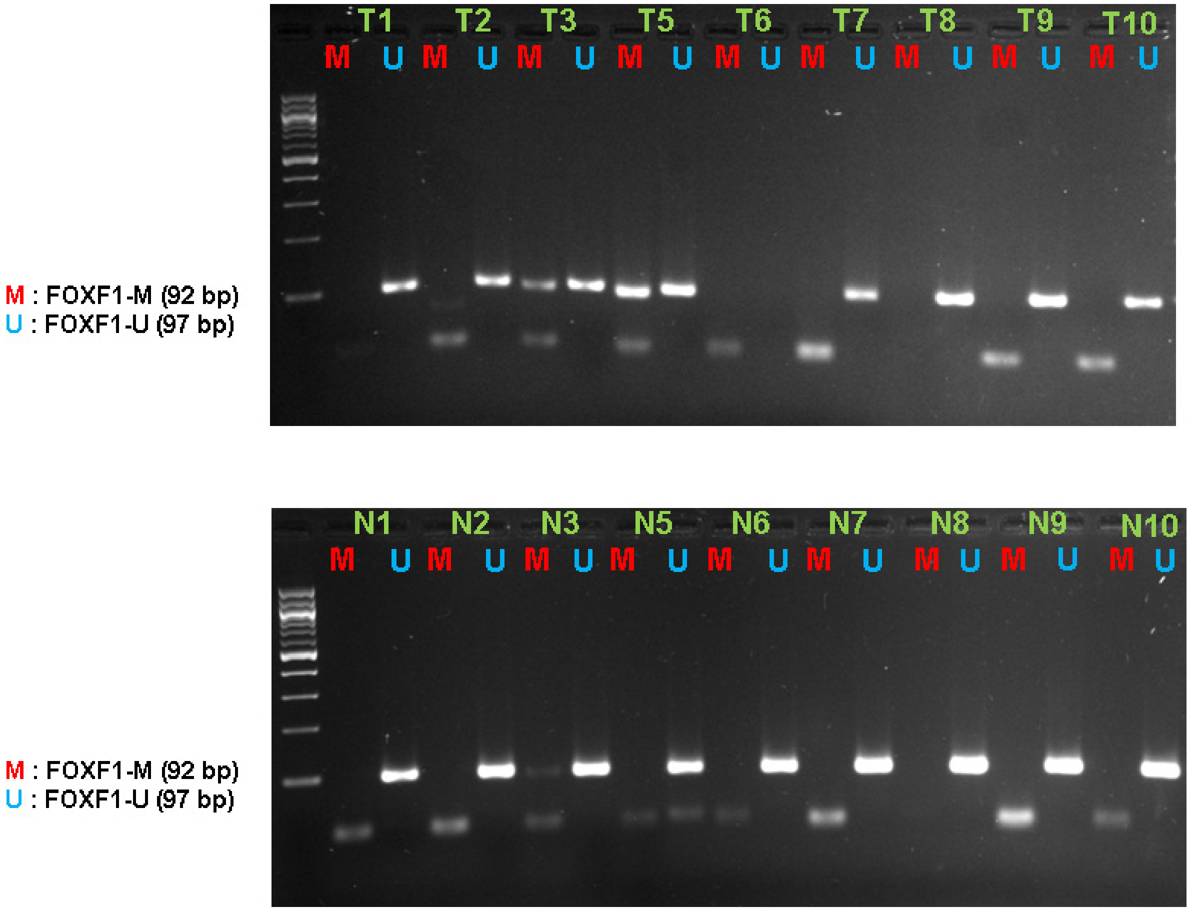

Supplementary Figure 4: Methylation-specific PCR (MSP) assay for the detection of *FOXF1* promoter hypermethylation in 10 lung adenocarcinoma samples (T) and their adjacent matched normal tissue (N).

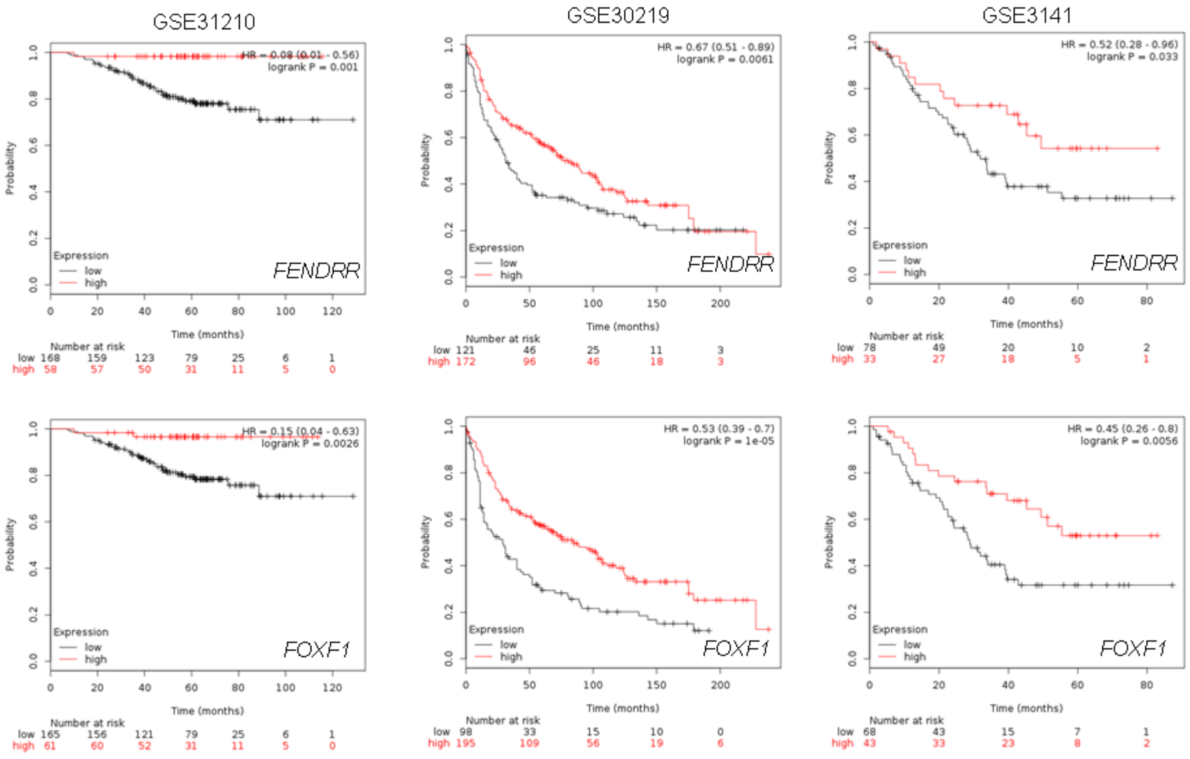

Supplementary Figure 5: Kaplan-Meier survival plots based on three different GSE arrays (GSE31210, GSE30219 and GSE3141) from lung cancer patients. Data were analyzed with the KM-plotter (http://kmplot.com/analysis/). Red and black lines indicate patients with high and low *FENDRR* and *FOXF1* expression respectively. The total number of patients in the two categories are shown at the bottom of the panel. Hazard ratios (HR) and p values (log rank p) are shown at the top of the panel.

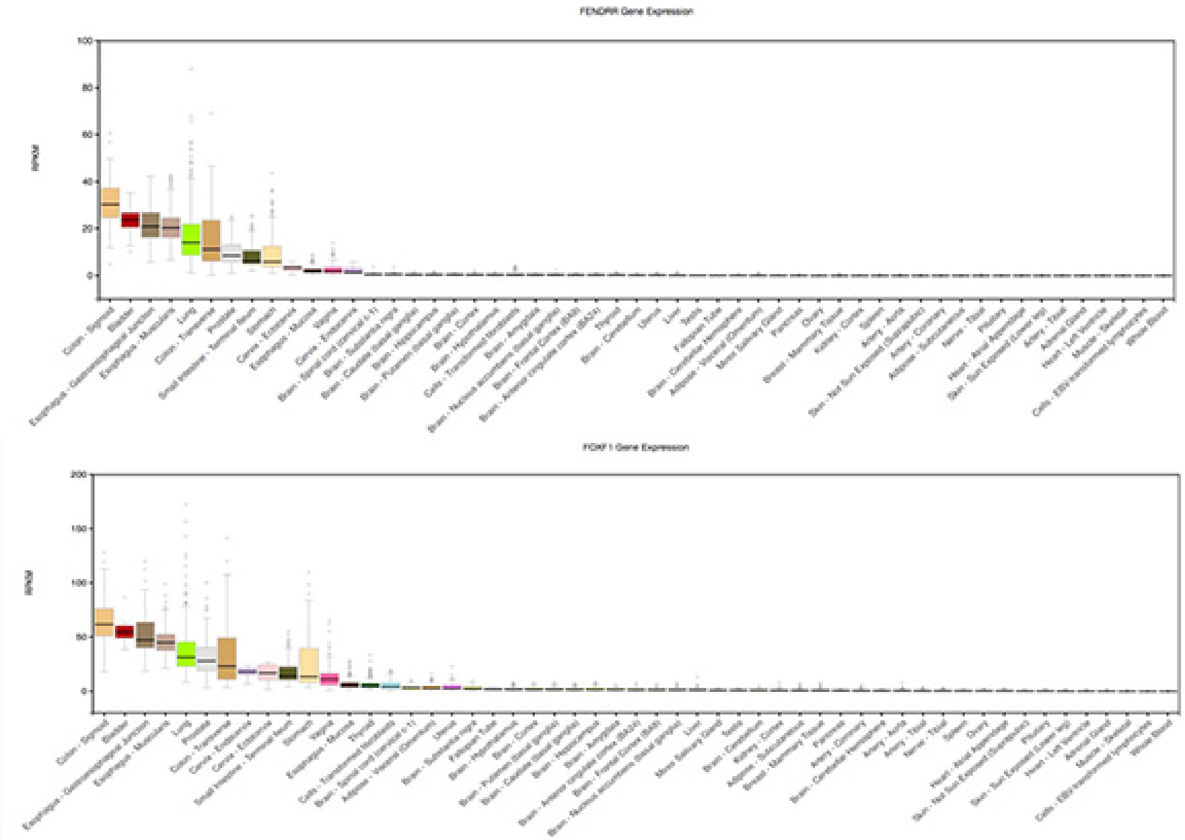

Supplementary Figure 6: *FENDRR* and *FOXF1* expression levels are relatively high in healthy lung tissue, with a median of 14 and 31 RPKM (Reads per kilo base per million of mapped reads) (n=320) respectively. Source: GTEx database.

Supplementary Table 1: Primers sequences using for qRT-PCR, pyrosequencing and MSP analysis

Primer qRT-PCR	
F-FOXF1	GATGTCTTCGGCGCCCGAGA
R-FOXF1	GGTAGGTGACCTGCTGGTGG
F-FENDRR	AGATCCTCCCGTGGAAGCC
R-FENDRR	CCTCAGAAATACACACCGGGG

Methylation assays
Pyrosequencing

Oligo Name	Oligo Sequence	Size	Sequence to analyze
FOXF1/F1	GGGTTGGTTGTATTTTT	208bp	TYGTTATYGG YGGTTATTTT TTT
FOXF1/R1Bio	[Btn]AACTCCACTCCTTTCCTTATA		
FOXF1/S1	GGGTTGGTTGTATTTTTG		
FOXF1/F2Bio	[Btn]GGGTTGGTTGTATTTTTGT	209bp	RAAAAAAACR AACRAAAACR CRAAAACRAA CRCRCCRCRA CTCRCTCRAA AAAAAT
FOXF1/R2	CAACTCCACTCCTTTCCTTATAAAA		
FOXF1/S2	ATCCAACAACTCCTC		

MSP

Oligon Name	Oligo Sequence	Size
FOXF1/M/F	GGTTTTGAGTTAATCGTCGGC	92bp
FOXF1/M/R	TTCTCCCGAACGAACCGC	
FOXF1/U/F	GGGGTTTTGAGTTAATTGTTGGT	97bp
FOXF1/U/R	ACTTCTCCCAAACAAACCAC	
